# Royal Hospital for Children and Women

**Published:** 1893-05-20

**Authors:** 


### ROYAL HOSPITAL FOR CHILDREN AND WOMEN.

The Bishop of Rochester presided at the festival dinner in aid of the Royal Hospital for Children and Women, in Waterloo Bridge Road, held at the Savoy Hotel on Monday, 15th inst. There was a fairly large gathering, and contributions amounting to £570 were announced during the evening.

The Bishop of Rochester, in proposing "The Royal Hospital for Children and Women," thought that occasionally in the history of such an institution it was well that someone who occupied an ecclesiastical position should take the chair, because no man could be brought face to face, as the clergy necessarily were, with a series of common homes in the ordinary round of life without feeling it not merely a duty, but a very high privilege, to advocate just such an institution as the Royal Hospital for Children and Women. It always seemed to him that the medical and ecclesiastical professions enjoyed in common one special privilege peculiar to themselves, in that they and they alone were brought into touch and contact with men and women in the real and deepest moments of their lives, when their needs were greatest, when their hearts were softest, and when they most required a word of sympathy. He ought by this time to be beginning to know something about the needs of South London, and he could assure them that there was no part of England in which those who were in suffering and sorrow were in that position more acutely, more numerously over a small area, or with greater need of assistance and help than was the case in the area which was beyond the river. There was a tendency nowadays, which they ought to be on their guard against—the extraordinary tendency to believe that an institution to be worthy of support must be a brand new one, and that institutions of any sort which had a long history behind them must necessarily be effete, and that therefore help must be transferred to something that began about six months ago. People seemed to have a prejudice against institutions which had a history. His Lordship believed, however, that the institution which (like the Royal Hospital for Children and Women) could look back over a long series of years of successful work and life was that to which men of experience would like to give their help rather than to one which was of the newest. (Hear, hear.) For the last four score years this hospital had commended itself better and better year by year to those who knew its work, and people might be sure that when they found an institution which had weathered many storms, and was still going forward day by day doing good and useful work, that that was the institution which most deserved support. (Applause.)

